# 68. Can you diagnose this condition on plain radiographs?

**DOI:** 10.1093/rap/rky034.031

**Published:** 2018-09-20

**Authors:** Madhurima Chetan, Ben Empson, Priyanka Chandratre

**Affiliations:** 1 Department of Rheumatology, Sandwell and West Birmingham Hospitals NHS Trust, Birmingham, UNITED KINGDOM; 2 Community Rheumatology Service, University of Birmingham, Birmingham, UNITED KINGDOM

**Introduction:** Tumoral calcinosis is a rare familial disorder of phosphate homeostasis associated with periarticular calcinosis. Multiple causative mutations have been identified in the literature, particularly in families of Middle Eastern and African ethnicity. Tumoral calcinosis typically presents in childhood and young adulthood with painless joint swelling. Enlarging periarticular deposits can cause complications such as restricted range of movement, nerve impingement and skin ulceration. We present an interesting case of a young female with recurrent episodes of oligo-arthritis. She was initially thought to have palindromic rheumatism, until plain radiographs of her hands and feet showed ectopic calcification at several joints. A diagnosis of tumoral calcinosis was made. This had a significant impact on patient care as immunosuppressive therapy was avoided.

**Case description:** A 23 year old teacher of South Asian ethnicity presented with episodes of flitting pain and swelling affecting metacarpophalangeal (MCP), proximal interphalangeal (PIP) and elbow joints. These episodes, lasting up to two weeks, first began aged 16 and had recently become increasingly frequent. In between episodes, she was completely asymptomatic and her activities of daily living were never compromised. There were no symptoms suggestive of connective tissue disease (CTD). There was no family history of note. She brought along photos of her hands during a flare, which showed clear synovitis at several MCP joints. On clinical examination, there were four tender and one swollen MCP joints, with a normal range of movement. General examination was unremarkable. Her bloods were as follows: ESR 30, CRP 3, RF negative, anti-CCP negative, ANA negative. Ultrasound imaging of the hands in clinic showed subclinical synovitis at four MCP joints. At this stage, the working diagnosis was palindromic rheumatism. She was started on naproxen for symptomatic relief and was offered the option of hydroxychloroquine or methotrexate if she continued to experience frequent episodes of arthritis. She was referred for formal ultrasound imaging of the hands, and X-ray imaging of the hands, feet and chest. Interestingly, the formal ultrasound was reasonably normal. The plain radiographs showed ectopic calcification around several small joints of the hands and feet together with benign calcified nodules at the left lung apex. The reporting radiologist suggested a diagnosis of tumoral calcinosis. More detailed re-evaluation of the patient’s history revealed that as a young child attending accident and emergency, she was told that radiographs showed ectopic calcification. This long history would be in keeping with familial tumoral calcinosis, rather than calcinosis secondary to an underlying CTD. Further investigation showed low adjusted calcium and vitamin D, but other bloods including PTH and phosphate were unremarkable. Her bone biochemistry normalised following vitamin D supplementation. After multidisciplinary team (MDT) discussion, tumoral calcinosis was considered the most likely diagnosis. Imaging appearances were of amorphous, cystic and lobulated calcification in a periarticular distribution. The MDT confirmed that this was distinct from bone malignancy and consistent with a diagnosis of tumoral calcinosis. Metabolic causes of calcinosis, such as chronic kidney disease, primary hyperparathyroidism and chronic tophaceous gout, were excluded by her normal biochemistry. Tumoral calcinosis is classically associated with high phosphate; normal phosphate should therefore raise suspicion of an underlying CTD. In our patient, CTD is less likely given the childhood history of calcinosis, absence of clinical features, and normal autoantibodies. It is important that her creatine kinase is checked at next review to exclude autoimmune myositis. Dystrophic calcinosis occurs in up to 40% of children and adolescents with dermatomyositis, although it is unusual in adults. The MDT decided that there was no indication for immunosuppressive treatment and that she may benefit from a referral to metabolic medicine. Reports in the literature describe treatment of symptomatic tumoral calcinosis with phosphate-lowering therapy and surgical excision, although recurrence of lesions is not uncommon.

**Discussion:** Imaging was invaluable in this case; ultrasound excluded synovitis and plain radiographs revealed calcinosis. The differential diagnosis for this radiographic appearance includes tumoral calcinosis, metabolic calcinosis, and dystrophic calcinosis in CTD. Therefore, good quality clinical information is essential for the radiologist to make the diagnosis. The expertise of the MDT was critical in selecting appropriate investigations to exclude other causes of calcinosis and reach a conclusive diagnosis of tumoral calcinosis. Making this diagnosis meant our patient could avoid unnecessary immunosuppressive treatment. The MDT approach will continue to be important for her future management, e.g. complementary therapy for warm wax baths, endocrinology for phosphate-lowering therapy, and orthopaedic surgery for resection of lesions. This case highlights the salient features of tumoral calcinosis. This is a rare condition affecting children and young adults. The clinical spectrum ranges from incidental radiographic findings, to swollen and restricted joints, to painful skin ulceration and secondary infection. The typical laboratory findings are high (or, less commonly, normal) phosphate, normal calcium, normal renal function and negative immunology. Plain radiographs show well-demarcated, lobulated calcification around joints. CT and MRI are useful if there is uncertainty. Classical CT appearances are of lobulated cystic calcifications communicating with the bursa. On T1-weighted MRI, tumoral calcinosis lesions show inhomogeneous low-signal intensity. Two patterns are observed on T2-weighted MRI: diffuse lower-signal, or nodular with alternating areas of high- and low-signal. A long-term follow-up study suggests overall prognosis in tumoral calcinosis is generally good. Our patient presented with episodic flitting joint pain and swelling; this does not exactly match the presentation of tumoral calcinosis described in the literature. Her presentation may be part of the broad clinical and genetic spectrum of tumoral calcinosis. However, it is also possible that tumoral calcinosis may represent an incidental finding in her case. She remains under follow-up.

**Key Learning Points:** Tumoral calcinosis is a familial disorder characterised by periarticular calcinosis. The MDT has an important role in the diagnosis and management of this rare condition. Imaging is valuable in the evaluation of joint pain and swelling when there is clinical uncertainty. Tumoral calcinosis has a characteristic appearance on plain radiographs. Other underlying causes for calcinosis, such as metabolic disease and CTD, must be excluded before diagnosing tumoral calcinosis. Immunosuppressive treatment is not needed in tumoral calcinosis.

**Disclosure: M. Chetan:** None. **B. Empson:** None. **P. Chandratre:** None.

